# A highly specific and sensitive nanoimmunosensor for the diagnosis of neuromyelitis optica spectrum disorders

**DOI:** 10.1038/s41598-019-52506-w

**Published:** 2019-11-06

**Authors:** Ariana de Souza Moraes, Doralina Guimarães Brum, Jéssica Cristiane Magalhães Ierich, Akemi Martins Higa, Amanda Stefanie Jabur Assis, Celina Massumi Miyazaki, Flávio Makoto Shimizu, Luís Antonio Peroni, M. Teresa Machini, Amilton Antunes Barreira, Marystela Ferreira, Osvaldo N. Oliveira, Fabio Lima Leite

**Affiliations:** 1 Institute of Tropical Medicine of São Paulo, University of São Paulo, São Paulo, São Paulo, 05403000 Brazil; 2 Department of Physics, Chemistry and Mathematics, Federal University of São Carlos, Sorocaba, São Paulo, 18052780 Brazil; 3 Nanoneurobiophysics research group (GNN), Federal University of São Carlos, Sorocaba, São Paulo, 18052780 Brazil; 4 Department of Neurology, Psychology and Psychiatry, São Paulo State University, Botucatu, São Paulo, 18618687 Brazil; 5 São Carlos Institute of Physics, University of São Paulo, São Carlos, São Paulo, 13560970 Brazil; 6 Rheabiotech Laboratory of Research and Development, Campinas, São Paulo, 13084791 Brazil; 7 Department of Biochemistry, Institute of Chemistry, University of São Paulo, São Paulo, 05508000 Brazil; 8 Department of Neurosciences and Behavioural Sciences, Ribeirão Preto Medical School, University of São Paulo, Ribeirão Preto, São Paulo, Brazil

**Keywords:** Diagnostic markers, Biosensors

## Abstract

A precise diagnosis for neuromyelitis optica spectrum disorders (NMOSD) is crucial to improve patients’ prognostic, which requires highly specific and sensitive tests. The cell-based assay with a sensitivity of 76% and specificity of 100% is the most recommended test to detect anti-aquaporin-4 antibodies (AQP4-Ab). Here, we tested four AQP4 external loop peptides (AQP4_61–70_, AQP4_131–140_, AQP4_141–150_, and AQP4_201–210_) with an atomic force microscopy nanoimmunosensor to develop a diagnostic assay. We obtained the highest reactivity with AQP4_61–70_-nanoimunosensor. This assay was effective in detecting AQP4-Ab in sera of NMOSD patients with 100% specificity (95% CI 63.06–100), determined by the cut-off adhesion force value of 241.3 pN. NMOSD patients were successfully discriminated from a set of healthy volunteers, patients with multiple sclerosis, and AQP4-Ab-negative patients. AQP4_61–70_ sensitivity was 81.25% (95% CI 56.50–99.43), slightly higher than with the CBA method. The results with the AQP4_61–70_-nanoimmunosensor indicate that the differences between NMOSD seropositive and seronegative phenotypes are related to disease-specific epitopes. The absence of AQP4-Ab in sera of NMOSD AQP4-Ab-negative patients may be interpreted by assuming the existence of another potential AQP4 peptide sequence or non-AQP4 antigens as the antibody target.

## Introduction

Neuromyelitis optica spectrum disorders (NMOSD) are inflammatory syndromes of the central nervous system (CNS), characterised by myelitis and optic neuritis events, which affect predominantly spinal cords and optic nerves^1^. Neuromyelitis optica (NMO) was classified as a subtype of multiple sclerosis (MS) for decades^2,3^, but it could be distinguished from MS after the NMO-IgG autoantibody was discovered in 2004, which was later identified as directed against the aquaporin-4 protein (AQP4)^4^. Lucchinetti *et al*.^5^ confirmed the pathological distinction among MS and NMO, and the role of autoantibodies against the aquaporin-4 protein (AQP4-Ab) in NMOSD immunopathology. The binding of AQP4-Ab to extracellular loops of AQP4 in astrocyte foot processes triggers a humoral immune response^6^, with the inflammation increasing the permeability of the blood-brain barrier and being likely to cause demyelination, axonal lesion, and necrosis^7^. The diagnosis of NMOSD has been based on clinical manifestations and magnetic resonance imaging at the optic nerve, spinal cord, brainstem, diencephalic and cortical regions, in addition to detection of the AQP4 antibody (AQP4-Ab) as in the cell-based assay (CBA) recommended by the International Panel for NMO Diagnosis^1,8^. CBA has an average sensitivity of 76% and specificity of 100%, thus failing to detect AQP4-Ab in 24% of the patients with NMOSD clinical manifestations^8^. This failure could be caused by: (i) undetectable serological levels of AQP4-Ab; (ii) reactivity with a different AQP4 sequence; or (iii) non-AQP4 antigen recognition^9–12^. New approaches with more sensitive methods are therefore needed for NMOSD diagnosis, which may include nanoimmunosensors such as those developed for detecting a biomarker for demyelinating diseases^13–17^. Indeed, sensors exploiting atomic force microscopy (AFM) may be sufficiently sensitive to diagnose patients for which AQP4-Ab is not detectable^18^. In this paper, we report an AFM nanoimmunosensor to detect interaction forces between samples of patients and AQP4 peptides, rather than with antigens as in the CBA method. Using peptides brings a series of advantages, mostly related to the simplicity in nanoimmunosensor assembly since it does not require protein expression on a cell surface. Also, the use of peptides allows for epitope mapping^19,20^. These molecules including immunogenic AQP4 peptides located on the astrocyte surface, known as loop A, loop C, and loop E, in NMOSD-related nomenclature were explored to identify AQP4 epitopes^20–22^. In order to verify which peptide would be specific for AQP4-Ab, we screened four AQP4 peptides from the extracellular loop (AQP4_61–70_, AQP4_131–140_, AQP4_141–150_, and AQP4_201–210_). These peptides were chosen because they are located in the extracellular regions of AQP4 protein^6^, where the interaction between AQP4-Ab and AQP4 protein is more likely to occur. The nanoimmunosensor assay permitted to identify AQP4_61–70_ as highly specific to distinguish NMOSD patients tested positive for AQP4-Ab from subjects, who were either seronegative for the AQP4-Ab or were not diagnosed with NMOSD.

## Results

### AFM screening of the peptide panel

**Figure 1:**
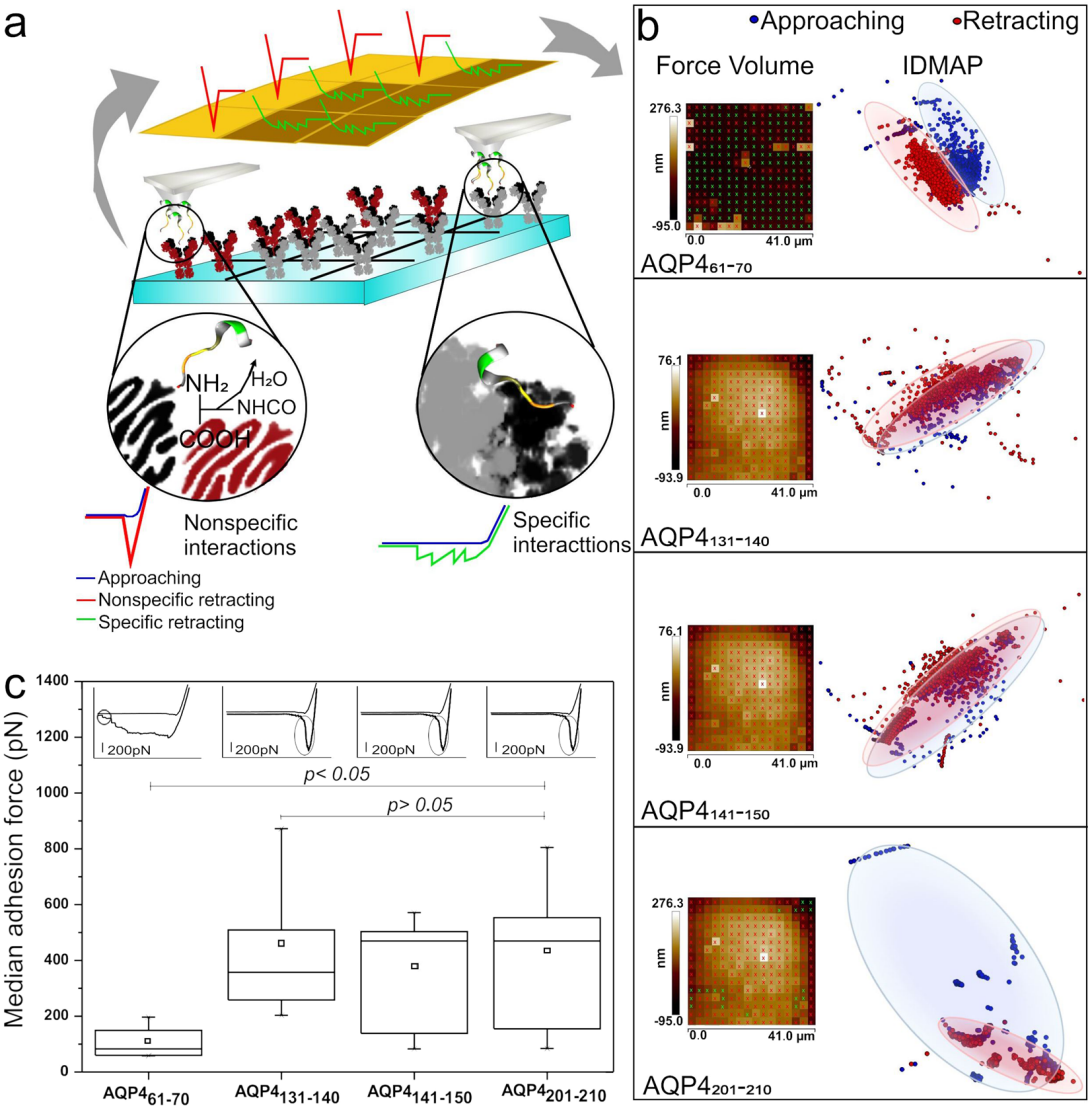
AQP4 peptides panel reactivity towards AQP4-Ab. (**a**) Force Volume scheme, which contained a nonspecific retracting curve (red) from amide bond formation (nonspecific interaction) or specific retracting curve (green) from Ag-Ab interaction. The blue line is the approaching curve. (**b**) IDMAP from Force Volume maps illustrating the prevalence of specific curves only for AQP4_61–70_. Imagens are 40 × 40 *μ*m^2^. The dark colour of pixels indicates smaller adhesion forces, while larger forces are shown as bright pixels. (**c**) Boxplot count quantifying adhesion forces from interactions among AQP4 peptides and AQP4-Ab; no significant difference was found between: AQP4_131–140_ and AQP4_141–150_ (*p* = 0.68); AQP4_131–140_ and AQP4_201–210_ (*p* = 0.79); AQP4_141–150_ and AQP4_201–210_ (*p* = 0.57). The interactions were significantly different among AQP4_61–70_ and other peptides (AQP4_131–140_, *p* = 0.0009; AQP4_141–150_, *p* = 0.02; and AQP4_201–210_, *p* = 0.007). The shapes of the representative curves are shown on the upper part of the graph, illustrating the prevailing interactions in each system. The region corresponding to the adhesion force is circled. The scale bar for the force represents 200 pN.

The first objective was to verify with atomic force spectroscopy (AFS) measurements whether one (or more) of the peptides had a specific interaction with the serum samples of NMOSD AQP4-Ab-positive patients tested with CBA. By specific interaction we mean a kind of Ag-Ab interaction, in contrast to nonspecific interactions deriving from nonspecific bindings that may include weak forces (e.g. hydrogen bonds or van der Waals forces)^23^ and covalent bonds in amide bond formation^24^. In principle, two factors can be used to distinguish between the two types of interaction: the intensity of the adhesion force and the shape of the approach-retracting curves. The adhesion forces for eight NMOSD AQP4-Ab positive patients were mapped with Force Volume, i.e. images where each pixel contains an approach-retracting force curve (in a total of 256 force curves per image). Two distinct patterns of force curves were identified, which differ especially in the retracting curve, as shown schematically in Fig. 1a: (i) retracting curves with only one slope are attributed to nonspecific interactions^25^; (ii) retracting curves with multiple repeated slopes are typical of specific interactions such as the Ag-Ab complex formation owing to hydrogen bonds and van der Waals forces^26,27^. Patterns of specific interaction were only observed when the AFM tip was coated with the AQP4_61–70_ peptide. This can be inferred from the interactive document mapping (IDMAP) plots^28^ in Fig. 1b, where a clear distinction between data for the retracting and approaching curves could be seen for AQP4_61–70_, but not for the other peptides. In this analysis, each curve was transformed into a single data point, marked in blue for the approaching and red for the retracting curves. The reason for the difference is that the approaching and retracting curves almost coincide for the nonspecific interactions, apart from a small region where there is a nonzero attractive force, while the differences are larger for the force curves associated with specific interactions (see Fig. 1a). Indeed, with specific interaction there is stretching or elongation of molecules throughout the retracting line, in contrast to the sharp detachment with nonspecific interactions. Another difference between the two types of force curves is in the intensity of the adhesion force. The Force Volume maps in Fig. 1b indicate a smaller force for the specific interactions with AQP4_61–70_. A more quantitative analysis was performed by selecting fifty spectra for each peptide, all of which had the overall behaviour for each class. This procedure of employing only part of the spectra was adopted because of the heterogeneity of the serum samples since not all the force curves presented the typical behaviour of their class. The boxplot graphs in Fig. 1c show that the median adhesion forces are practically indistinguishable for the peptides AQP4_131–140_, AQP4_141–150_ and AQP4_201–210_ peptides. In contrast, the median adhesion forces were significantly distinct for AQP4_61–70_ compared to the other peptides (AQP4_131–140_, *p* = 0.0009; AQP4_141–150_, *p* = 0.02, and AQP4_201–210_, *p* = 0.007). Note the smaller median adhesion force (85.50 pN; IQR 68.50–160.50) for the specific interaction with AQP4_61–70_, to be contrasted with 416.25 pN (301.60–591.25), 470.00 pN (138.50–503.50), and 489.10 pN (198.70–604.90) for AQP4_131–140_, AQP4_141–150_, and AQP4_201–210_, respectively. The larger forces for nonspecific interactions are mostly likely due to covalent bonds in amide formation^24^, which are prevented by the Ag-Ab type of interaction due to bioaffinity of this complex (affinity and avidity)^29,30^ in the case of AQP4_61–70_. Furthermore, the adhesion force values measured for AQP4_61–70_ are consistent with those reported between antigens and antibodies using the AFS technique^18^. Because a smaller adhesion force for a specific interaction seems counterintuitive, we performed a series of subsidiary AFS experiments at various pHs, whose results are given in the Supplementary Information. The analysis confirms the hypothesis above to explain the stronger adhesion forces for the peptides with nonspecific interactions.

### AQP4_61–70_-nanoimmunosensor as NMOSD diagnostic tool

**Figure 2:**
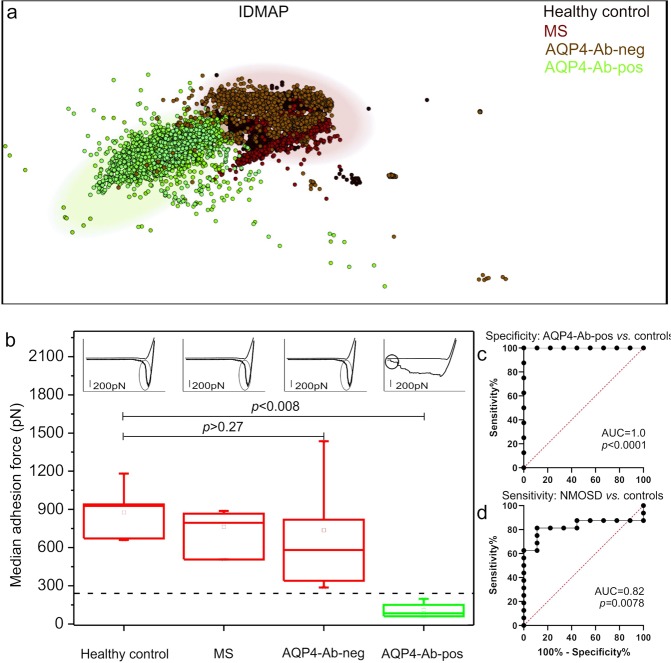
Performance and accuracy of the AQP4_61–70_-nanoimmunosensor. (**a**) IDMAP plot for the retracting curves showing distinct clusters for the specific and nonspecific interactions in NMOSD (AQP4-Ab-positive, in green) and negative control (healthy, in black)/MS (dark red)/AQP4-Ab-negative (brown). (**b**) Box plot quantifying median adhesion forces obtained with the AQP4_61–70_-nanoimmunosensor for the samples of healthy control, MS, AQP4-Ab-negative, and AQP4-Ab-positive. The AQP4_61–70_-nanoimmunosensor was effective in distinguishing AQP4-Ab-positive from other samples (healthy control, *p* = 0.004; MS, *p* = 0.008; AQP4-Ab-negative, *p* = 0.0009). The shapes of the representative curves are shown on the upper part of the graph, illustrating the prevailing interactions in each system. The region corresponding to the adhesion force is circled. The scale bar for the force represents 200 pN. (**c**) ROC curve for the comparison between AQP4-Ab-positive and controls (healthy volunteers and MS), with the AUC value of 1.0, confirming the high specificity of the AQP4_61–70_-nanoimmunosensor assay, with *p* = 0.0001. The resulting cut-off was 241.3 pN at 100% of specificity (95% CI 63.06–100). (**d**) ROC curve for the comparison between AQP4-Ab-positive and -negative patients with healthy control and MS with AUC value of 0.82 with *p* = 0.0078, resulting in 81.25% sensitivity (95% CI 56.50–99.43) for the AQP4_61–70_-nanoimmunosensor assay.

The distinctive interaction between AQP4_61–70_ and the serum samples of AQP4-Ab-positive patients in Fig. 1 motivated us to test the nanoimmunosensor for diagnosis of NMOSD, which means being selective for AQP4 and without false positives when samples from non-NMOSD patients are considered. We used seventeen purified IgGs samples of the serum: five of healthy volunteers (negative control), four MS, and eight NMOSD AQP4-Ab-negative patient samples to compare with adhesion forces for NMOSD AQP4-Ab-positive patients. The IDMAP plot in Fig. 2a obtained from the raw data for the retracting curves of all samples shows a clear separation between the NMOSD AQP4-Ab-positive patients and the other subjects. This separation is better seen in the median adhesion forces in Fig. 2b, which are 927 pN (IQR 672–937), 831 pN (650.50–877.50) and 605 pN (425–1055.70), for the healthy control, MS, and AQP4-Ab-negative samples, respectively, much larger than the 85.50 pN (68.50–160.50) for the AQP4-Ab-positive samples. The specificity of the assay with the AQP4_61–70_-nanoimmunosensor was tested with the receiver operating characteristic (ROC) curve to analyse the accuracy in distinguishing the healthy volunteers and MS patients from NMOSD AQP4-Ab-positive patients. Figure 2c presents the ROC curve in which the AQP4_61–70_-nanoimmunosensor was effective in discriminating AQP4-Ab-positive from controls (healthy volunteers and MS) with an AUC value of 1.0, with *p* < 0.0001. The assay sensitivity was tested with ROC curve to verify if AQP4-Ab-negative would be distinguished from AQP4-Ab-positive NMOSD patients. Figure 2d displays the ROC curve resulting in an AUC value of 0.82, with *p* = 0.0078, which proved that these groups are distinct. The cut-off value of 241.3 pN was determined from the ROC curve in Fig. 2c, at 100% of specificity (95% CI 63.06–100) with the AQP4_61–70_-nanoimmunosensor assay. The sensitivity of this diagnostics assay was 81.25% (95% CI 56.50–99.43) (Fig. 2d). Therefore, adhesion forces below this cut-off threshold using the AQP4_61–70_-nanoimmunosensor indicate the presence of AQP4-Ab.

## Discussion

The peptide sequence AQP4_61–70_ (GTEKPLPVDM) from the extracellular loop of AQP4 was found to bind specifically to the serum samples of NMOSD patients tested positive for AQP4-Ab, which is in contrast to Kampylafka *et al*.^20^ who reported major AQP4-Ab reactivity against AQP4 intracellular loops. However, this specific binding is consistent with recent studies where extracellular loops referred to as loop A, loop C, and loop E were found as disease-specific epitopes for NMOSD diagnosis^19,21,22^. AQP4_61–70_ is contained in loop A, thus demonstrating the role of this sequence as an epitope of AQP4 protein due to its high reactivity with AQP4-Ab. Although some studies with whole protein reported that AQP4 conformation can interfere in the recognition by AQP4-Ab^31,32^, other approaches using peptides seem to be promising to understand the heterogeneity of NMOSD AQP4-Ab-negative patients with regard to which sequence of AQP4 or other non-AQP4 antigens is responsible for the pathology. The performance of the AQP4_61–70_-nanoimmunosensor is higher than for most published sensors^33^, including those with the CBA assay. The sensitivity of CBA can be related to the intrinsic sensitivity of the method or to the AQP4-Ab absence against the AQP4_61–70_ sequence in the NMOSD AQP4-Ab-negative patient group. With the nanoimmunosensor strategy reported here, it is possible to identify new AQP4 peptide sequences (see Fig. S2; Supplementary Information) to expand the AQP4 peptide panel and address a crucial issue involving CBA: should the researchers try to improve the assay sensitivity or patients absolutely do not have AQP4-Ab? This issue has attracted interest because the meaning of the AQP4-Ab absence is still unknown. It is possible that AQP4-Ab-negative patients have an antigen typical of another pathology instead of MS or NMOSD, for example that could bind to the antibody against the myelin oligodendrocyte glycoprotein (MOG-Ab). A percentage of acute disseminated encephalomyelitis (ADEM) and NMOSD AQP4-Ab-seronegative patients were seropositive to MOG-Ab^34^. Though approximately 20% of NMOSD AQP4-Ab-negative were seropositive to MOG-Ab^11^ and there was evidence that this antibody was related to relapses^35^, there is another hypothesis, as follows. Based on Nakashima^36^, it would be inappropriate to include AQP4-Ab-negative and MOG-Ab-positive patients in NMOSD or ADEM categories. Moreover, the NMOSD AQP4-Ab-negative phenotype may refer to other antigens targeted by a distinct mechanism^37^, as observed in the autoimmune disorder myasthenia gravis (MG) for which the acetylcholine receptor (AchR) antibody was found to be the disease’s biomarker. Other targets were found as disease-specific epitopes for distinct MG phenotypes, as the muscle-specific kinase (MuSK) protein and the lipoprotein-related protein 4 (LRP4). Antibodies against these proteins were identified in MG AChR-seronegative patients^38^. The same may apply to AQP4-Ab-negative patients, i.e. different disease-specific targets might exist in the mechanisms responsible for NMOSD. One should also stress an implication of the findings here. Sequences of amino acids, e.g. peptides, can participate in autoimmune diseases as immunogenic sequences, with binding sites composed of linear epitopes^39^.

## Methods

### Patients and samples

**Figure 3:**
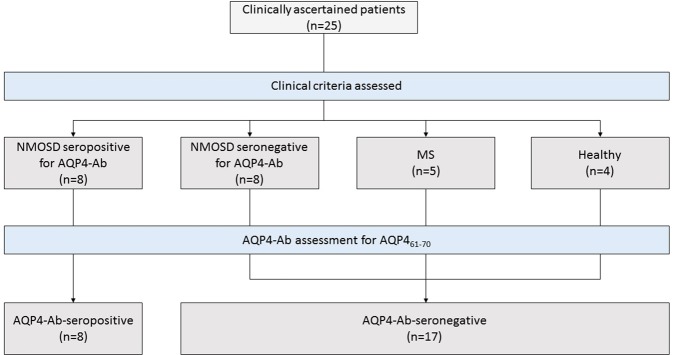
Trial profile.

All patients involved in this research provided written informed consent after they were informed about the study and any associated risks. All experiments were conducted according to the Declaration of Helsinki, in compliance with laws and institutional guidelines, and approved by: (a) Ethics Committee of University of São Paulo Medical School, Brazil, under the Certification of Ethical Presentation and Approval CAAE number 51297215.3.3001.0065; (b) Ethics Committee of Federal University of São Carlos, Brazil, under the Certification of Ethical Presentation and Approval CAAE number 51297215.3.0000.5504; (c) Ethics Committee in Research of Medical School of Botucatu, São Paulo State University, Brazil under the Certification of Ethical Presentation and Approval CAAE number 51297215.3.3002.5411. From August 30, 2015, to December 1, 2017, we enrolled in this study a total of twenty-five subjects (see Fig. 3). A cohort of sixteen NMOSD patients was diagnosed according to the diagnostic criteria, convened by the International Panel for NMO Diagnosis^1^. Patients 1 through 8 were AQP4-Ab-positive, and 9 through 16 were AQP4-Ab-negative based on previous tests with CBA at the moment of their diagnosis. In addition, nine other subjects’ samples were used: five from healthy volunteers and four from MS patients. The serum samples were purified by protein G affinity chromatography (protein G sepharose, GE healthcare) and quantified by the Bradford method^40^ at Rheabiotech Development, Production and Commercialisation of Biotechnology Products, Ltd., Campinas, Brazil.

### Surface functionalisation

AFM rectangular cantilevers of silicon nitride (AC40, Bruker, Nano Inc., Billerica, USA) were used with spring constant (*k*) of 0.02 N.m^−1^ and tip radius of 8 nm, suitable for force curve measurements^41^, and mica sheets (TedPella Inc., Redding, USA) of 15 × 15 mm^2^ were cleaned in UV/Ozone radiation (ProCleaner^TM^, UV.PC.220, Bioforce Nanosciences, Ames, USA) during 20 min. Both surfaces were aminated with 3-aminopropyltrietoxisilane (APTES) and triethylamine (Sigma-Aldrich®, St. Louis, USA), following an established protocol^13,42,43^.

### Molecules immobilisation

#### Nanoimmunosensor

AFM cantilevers were covered with a bifunctional PEG solution (NH_2_–PEG–COOH, average Mn of 2000; 0.01 *μ*g.mL^−1^; Sigma-Aldrich®, St. Louis, USA) to provide carboxyl end groups on the surface followed by immobilisation of the peptide. Each of the four peptides (AQP4_61–70_: GTEKPLPVDM, AQP4_131–140_: GILYLVTPPS, AQP4_141–150_: VVGGLGVTMV, AQP4_201–210_: SMNPARSFGP; 0.1 *μ*g.mL^−1^; Peptide Chemistry Laboratory of the Institute of Chemistry, São Paulo, Brazil, and Genscript, Piscataway, USA) was immobilised with 1-ethyl-3-(3-dimethylaminopropyl)carbodiimide hydrochloride (EDC, 0.4 M) and N-hydroxysuccinimide (NHS, 0.1 M) (Sigma-Aldrich®, St. Louis, USA) to activate PEG carboxyl end groups to interact with peptides primary amine end groups.

#### Substrate

Protein A solution (30 *μ*L, 0.01 *μ*g.mL^−1^) was dropped on mica sheets (15 × 15 mm^2^, Ted Pella Inc, Redding, California, USA) to yield the immobilisation of suitably oriented IgGs^44^. Then, a solution with IgGs purified from the NMOSD patients’ serum samples (30 *μ*L, 56 *μ*g.mL^−1^) was dropped on mica sheets. The samples were taken from eight patients with NMOSD AQP4-Ab-negative serologic status, eight patients with NMOSD AQP4-Ab-positive serologic status, four patients diagnosed with MS and five healthy volunteers.

### Measurement acquisition

Force curves were obtained with a Bruker AFM, MultiMode V controller (Veeco Instruments Inc, Plainview, USA), and a Picoforce package. All measurements were performed in a fluid cell with Milli-Q® water using a loading rate of 184 nN.s^−1^. Adhesion forces were extracted from the force curves to evaluate the interactions between each of four peptides (AQP4_61–70_, AQP4_131–140_, AQP4_141–150_, and AQP4_201–210_) and eight AQP4-Ab-positive serum samples from NMOSD patients.

Then, forces were measured using the nanoimmunosensor made with AQP4_61–70_ peptide for eight AQP4-Ab-negative NMOSD patients, MS, and healthy volunteers. Measurements were analysed using Nanoscope Analysis 7.30 and Origin 8.0 software. The Force Volume technique was applied to obtain 256 measurements (Fig. S1; Supplementary Information) of each serum sample and then fifty measurements from specific interactions were selected to be analysed quantitatively, according to the method reported by Bizzarri and Cannistraro^27^.

### Statistical analysis

Results were analysed with the boxplot graph due to the nonparametric characteristic of our data. The U-test Mann Whitney was applied to determine p values for assessing statistical differences. The nanoimmunosensor accuracy was analysed with the receiver operating characteristic (ROC) curve, which determines *p* value, cut-off, and area under the ROC curve (AUC).

In addition, ROC was used to analyse sensitivity and specificity of the nanoimmunosensor, i.e., the nanoimmunosensor efficiency in distinguishing typical NMOSD patients from a set of AQP4-Ab-negative, MS patients, and healthy volunteers (n = 25 measured in triplicate), as well as the presence or absence of AQP4-Ab in the patients’ serum samples.

### Data treatment with information visualisation

Raw adhesion force (pN) vs. position (nm) spectra were analysed with multivariate data analysis using the PEx-Sensors software. The dissimilarities between the samples were converted to Euclidean distances. Because of the high dimensionality of the data (462 dimensions), they were reduced to a two-dimensional representation with the algorithm Fastmap and further improved with the Force Scheme algorithm using 500 iterations to recover some of the lost precision during data reduction. Mapping was performed with the Interactive Document Map (IDMAP) technique^45^, which has been successful in the analysis of biosensing data^46–48^.

### Surface plasmon resonance

Surface plasmon resonance (SPR) measurements were carried out via the BioNavis SPR Navi 200 system with a sensing device (50 nm-thick gold layer covered glass slides) previously cleaned in a mixture of 5H_2_O:1H_2_O_2_:1NH_4_OH (v/v) for 10 min at 85 °C.

Glass slides were aminated with cysteamine (1.92 mg.mL^−1^), and functionalised as follows: (i) PEG immobilisation, (ii) peptide immobilisation, and (iii) antibody detection. In each cycle the coated slides were washed extensively with Milli-Q® water. The wavelength used was 670 nm in a Kretschmann configuration^49^.

### Characterisation of AQP4_61–70_-nanoimmunosensor

In subsidiary experiments we employed the SPR technique^50^ to verify the molecular architecture assumed to be valid for the AFM AQP4_61–70_-nanoimmunosensor, and confirm that a nanoimmunosensor can be made with another principle of detection. Two SPR channels were used for injections at the same time, which differ only in the last step: one with an injection of Milli-Q® water flow as the negative control (reference channel) and the other with antibodies flow (detection channel).

**Figure 4:**
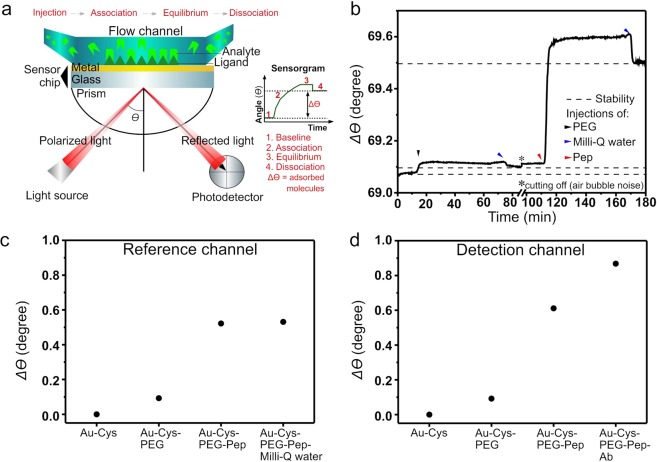
Characterisation of the functionalisation process and AQP4-Ab detection by SPR. (**a**) SPR operation. (**b**) Adsorption kinetics for PEG and peptide injections. (**c**) and (**d**) Comparison between reference channel and sensor application (detection channel) with AQP4-Ab detection.

The sensorgram illustrates the resonance angle extracted from the kinetic parameters of the sensor assembly steps in real time (Fig. 4a). The adsorption of the polyethylene glycol (PEG) crosslinker on the aminated surface with cysteamine is depicted in Fig. 4b in which an angle shift Δ *θ* of 0.09° was obtained in both the reference and detection channels. Adsorption of peptide molecules on the PEG layer led to an angle shift Δ *θ* of 0.43° and 0.51° in reference and detection channels, respectively (Fig. 4b).

By comparing with the results for the negative control (Milli-Q® water flow), one infers from Fig. 4c,d that there is antigen (AQP4_61–70_ peptide) recognition by AQP4-Ab, noticed by Δ *θ* 0.01° and Δ *θ* 0.26°, respectively. The changes in resonance angle are presented in Table 1.

**Table 1:** Resonance angles in the functionalisation steps and AQP4-Ab detection.

Steps	Reference channel			Detection channel		
	Initial	Final	Δ *θ*	Initial	Final	Δ *θ*
Au-Cys	0°	0°	—	0°	0°	—
Au-Cys-PEG	0°	0.09°	0.09°	0°	0.09°	0.09°
Au-Cys-PEG-Pep	0.09°	0.52°	0.43°	0.09°	0.60°	0.51°
Detection of AQP4-Ab	0.52°	0.53°	0.01°	0.60°	0.86°	0.26°

The molecules persistence on the surface after washing with Milli-Q® water flow produce Δ *θ* values^51,52^, as observed here. According to Janmanee *et al*.^53^, each adsorption step occurs by covalent linkages. Here, in the first step adsorption was due to amide II formation between NH_2_ group of Cys and COOH group of PEG. The same amide II group was formed between NH_2_ of PEG and COOH of AQP4_61–70_ peptide. The increase in the angle in the sensorgram when comparing Δ *θ* of the reference channel with the detection channel pointed to AQP4-Ab binding to AQP4_61–70_ peptide, as expected from other studies^54–56^.

## Supplementary information

A highly specific and sensitive nanoimmunosensor for the diagnosis of neuromyelitis optica spectrum disorders

## Data Availability

All data that were generated or analysed during this study and that supports the reported findings are included in this paper and additionally provided as supplementary information.
