# Supplementary material for: A highly specific and sensitive nanoimmunosensor for the diagnosis of neuromyelitis optica spectrum disorders
Source: Sci Rep. 2019 Nov 6;9:16136. doi: 10.1038/s41598-019-52506-w (PMC6834626; doi:10.1038/s41598-019-52506-w)
Supplement: Supplementary file 1 — A highly specific and sensitive nanoimmunosensor for the diagnosis of neuromyelitis optica spectrum disorders [file 41598_2019_52506_MOESM1_ESM.pdf]

Supplementary Information

Title: A highly specific and sensitive nanoimmunosensor for the diagnosis of neuromyelitis optica spectrum disorders

Authors: Ariana de Souza Moraes, Doralina Guimarães Brum, Jéssica Cristiane Magalhães Ierich, Akemi Martins Higa, Amanda Stefanie Jabur Assis, Celina Massumi Miyazaki, Flávio Makoto Shimizu, Luís Antonio Peroni, M. Teresa Machini, Amilton Antunes Barreira[‡], Marystela Ferreira, Osvaldo Novais Oliveira Jr, Fabio Lima Leite*

*Author for correspondence.

[‡]Deceased in November 14th, 2018.

Supplementary information

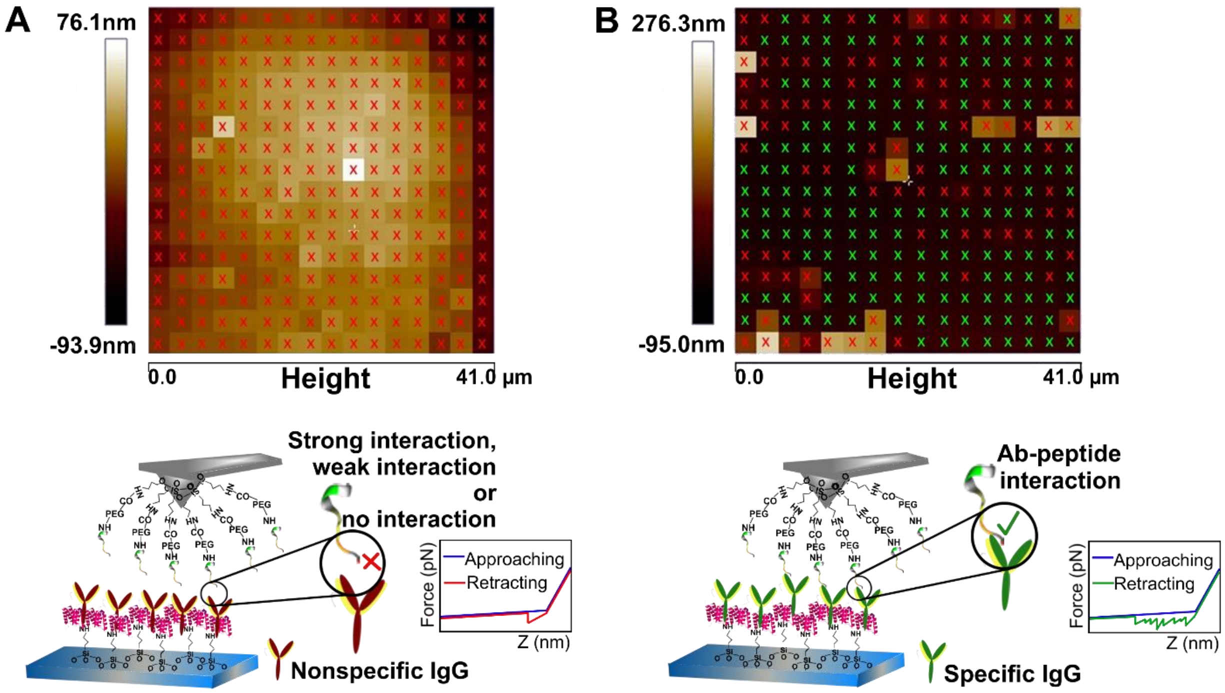

Figure S1: Map of adhesion forces with the Force Volume technique. (A) Nonspecific interactions from weak or covalent bindings, evidenced by only one slope in the retracting force curve. (B) Specific forces from antigen-Ab interactions, resulting in a force curve with multiple repeated slopes in the retracting line. (IgG, immunoglobulin).

Intracellular and extracellular peptides

A part of the same peptide used by Kampylakfa and colleagues [1] ($AQP4_{1-10}$: MSDRPTARRW) was immobilized on AFM tip. This nanoimmunosensor interacted with AQP4-Ab-negative samples from AQP4-Ab-seronegative NMOSD patients and healthy volunteers.

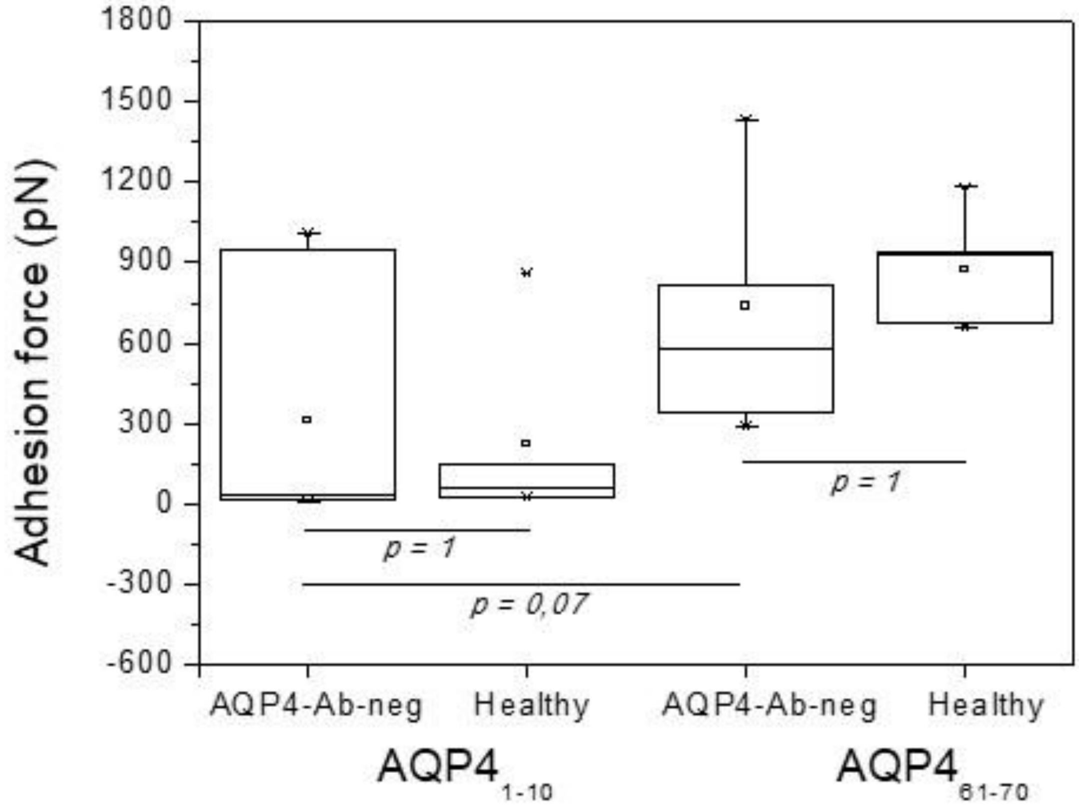

Figure S2: Comparison between the interaction of AQP4-Ab-seronegative patients and healthy serum samples with intracellular ($AQP4_{1-10}$) and extracellular peptides ($AQP4_{61-70}$) immobilized on the AFM nanoimmunosensor.

The results show that peptide $AQP4_{1-10}$ and $AQP4_{61-70}$ are not the specific epitope of NMOSD seronegative phenotype. These findings are preliminary data of undergoing analyses of our work.

pH analyses

Supplementary methods

To analyse the biological systems at different pHs, the AFM tip was functionalised with $AQP4_{61-70}$ peptide. Substrates were functionalised with AQP4-Ab-negative and AQP4-Ab-positive samples. The measurements were performed in phosphate buffer of pH 7.4, 2.0, 11.0 and then the substrates used in these two latest media were re-analysed at pH 7.4, as follows:

Table S1: Analysed systems description.

pH	Samples	
**2.0**	AQP4-Ab-positive	AQP4-Ab-negative
**7.4**	AQP4-Ab-positive	AQP4-Ab-negative
**11.0**	AQP4-Ab-positive	AQP4-Ab-negative
**7.4**	AQP4-Ab-positive (re-analysis, after pH 2.0)	AQP4-Ab-positive (re-analysis, after pH 11.0)

The cutoff values (maximum and minimum threshold) were calculated from the ROC curve. The *p* values were calculated with the nonparametric U-test of Mann-Whitney.

Supplementary discussion: Experiments in different pH conditions resulted in adhesion force values for nonspecific interactions lower or higher than the specific interaction, depending on the media ionic condition.

Electrostatic and chemical forces involved in the specific and nonspecific systems

For the investigation of molecular events in the nanoimmunosensor measurements, especially concerning the lower adhesion force values for specific interactions (AQP4-Ab-positive) in comparison with nonspecific interactions (AQP4-Ab-negative), additional measurements at different pH values were carried out using AFM. The same protocol described in the Methods section was used for the tip and substrate functionalisation, and pH values of 7.4, 11.0, and 2.0 were considered for the fluid cell medium in the AFM experiments.

Case 1: the adhesion forces are lower in the nonspecific system than in the specific system. At pH 7.4, the median adhesion forces of AQP4-Ab-positive system was 160 pN (IQR 116.60–223.50). At pH 2.0, the median adhesion force values in the AQP4-Ab-positive and AQP4-Ab-negative systems were 47 pN (IQR 28–60.60) and 39 pN (17.90–57), respectively (figure S3). At pH 2.0, the antibody is denatured [2] by hydrogen bonds breakage [3] and the positively charged peptide tends to fold and agglomerate [4] as its isoelectric point is 3.93 [5] (figure S4). Hence, no Ag-Ab interaction force was observed, only low values of adhesion forces which resulted from hydrogen bonds [6,7] (figure S4). Experiments showed that changes in the

antibodies and peptides structure occurred, altering the predominant interaction and, consequently, generating distinct adhesion forces under different pH conditions.

Case 2: the adhesion forces are higher in the nonspecific system than in the specific system. At pH 7.4, the median adhesion forces of AQP4-Ab-positive and AQP4-Ab-negative systems were 160 pN (IQR 116.60–223.50) and 926 pN (659–1180), respectively. At pH 11.0, the median adhesion forces in the AQP4-Ab-positive and AQP4-Ab-negative systems were 633 pN (559.50–692) and 898 pN (700.90–1071), respectively. After the measurements performed at pH 2.0 and 11.0 and re-analysed at pH 7.4, the median adhesion forces for AQP4-Ab-positive were 840 pN (628–994.80) and 652 pN (518.70–955), respectively (figure S3). The pH values of 7.4 and 6.2 are optimal to Ag-Ab (antigen-antibody) interactions [2], thus the occurrence of specific interactions were reflected in the lowest adhesion forces values (figure S3), which are the outcome of the inherent hydrogen bonds, electrostatic, and van der Waals forces from the antigen recognition by the specific antibody [8] (figure S3). When the AFS measurements were performed with samples that do not contain the specific antibody, the Ag-Ab interaction was absent and other forces prevailed, as the covalent linkages and electrostatic interaction [9], mainly the latter because at pH 7.4 the $AQP4_{61\text{-}70}$ peptide is negatively charged [5] and the antibody is positively charged [2] (figure S4). In addition, at pH 7.4 (as well as at pH 6.2), the adhesion forces in the nonspecific system were higher than in the specific system (figure S3), which resulted from the covalent linkages [9] in amide bond formation between the available peptide amine I and the antibody carboxyl end groups (figure S4). At pH 11.0, there was no interaction between Ag-Ab (antibodies are denatured) [2], and the highest forces were also resultant from covalent linkages (figure S3). At pH 7.4 succeeding the measurements at pH 2.0 and pH 11.0, *i.e.* after denaturation of antibodies, the loss of peptide biological activity due to hydrolysis [10] yielded the highest adhesion forces (figure S3) as a consequence of amide bond formation between the available amine I of the peptide and the antibody carboxyl end groups (figure S4).

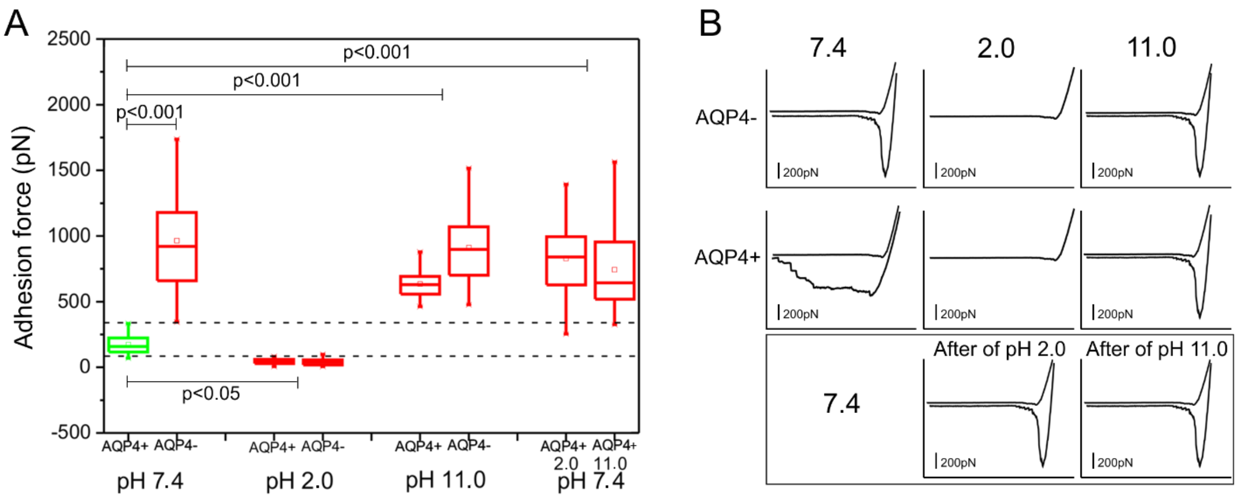

Figure S3: Adhesion forces at different pH values. (A) Cutoffs (dashed lines) calculated from the ROC curve were 84 pN (minimum threshold) and 339 pN (maximum threshold). At pH 7.4, specific interactions were distinguished from the nonspecific interactions, with $p<0.001$. At pH 2.0, adhesion forces are below the minimum threshold, concerning specific interactions, $p<0.05$. At pH 11.00, adhesion forces are above the maximum threshold, concerning specific interactions, $p<0.001$. At pH 7.4 after antibody denaturing at pH 2.0 and 11.0, adhesion forces are above the maximum threshold for specific interactions, $p<0.001$. At pH 7.4, AQP4-Ab-positive system was the only one with curve shape characteristic of a specific interaction. For all the other systems, the curve shapes were characteristic of nonspecific interactions: at pH 2.0, the force curves were typical of weak interactions but curves typical of strong interactions were observed when the same samples were re-analysed at pH 7.4. (B) Curve shapes.

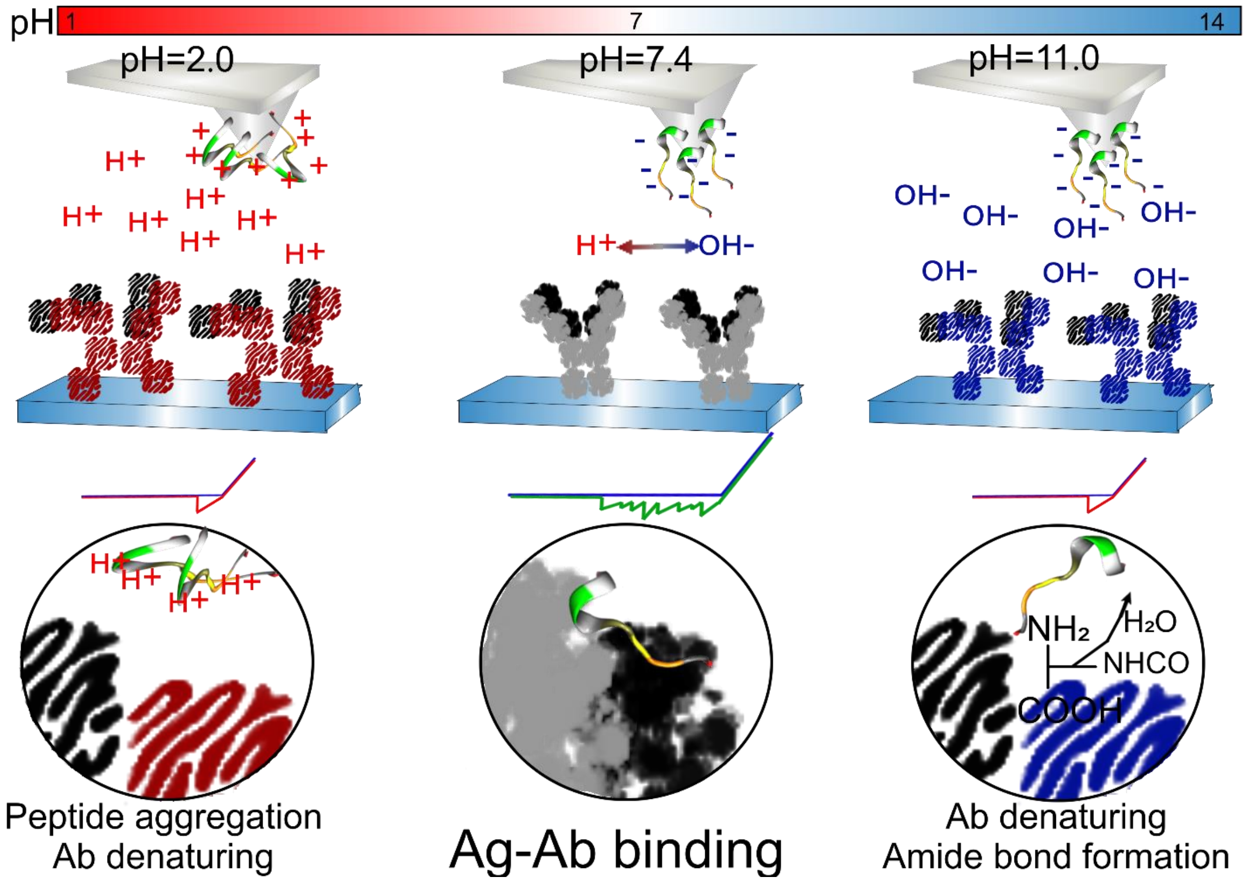

Figure S4: Molecules behaviour at different pH. At pH 2.0, antibody denaturing occurs because of hydrogen bonds breakage and structural changes. In addition, peptides tend to aggregate owing to hydrogen bonding, for the analyses were conducted below the peptide isoelectric point (which is 3.93). The optimal pH for Ag-Ab interaction is 7.4. At pH 11.0 no Ag-Ab interaction occurs due to antibody denaturing, thus covalent bonding appears with amide bond formation.

Table S2: Median adhesion forces for interaction between AQP4-Ab-positive or AQP4-Ab-negative serum samples with $AQP4_{61\text{-}70}$.

NMOSD serologic status	Subject	Adhesion forces (pN)
		Median (IQR)
**AQP4-Ab-positive**	1	149.50 (94–239)
	2	83 (48–118)

	3	88 (55–160)
	4	60 (34–94)
	5	77 (57–107)
	6	58.50 (37–112)
	7	196.50 (111–309)
	8	171.50 (111–282)
**AQP4-Ab-negative**	9	511.50 (409–727)
	10	286 (221–422)
	11	1436 (491–902)
	12	580.50 (439–1158)
	13	339 (243–533)
	14	1293 (755–1907)
	15	629.50 (389–1244)
	16	818.50 (488–1455)
**MS**	17	506 (205.50–1065)
	18	888 (728–989.50)
	19	795 (637.90–981.60)
	20	867 (737.80–1050)
**Healthy volunteers**	21	1181 (989–1305)
	22	672 (595–762)
	23	658.50 (498–838)
	24	939 (689–1065)
	25	927 (774–1059)

Data are median (IQR) in picoNewtons (pN). NMOSD, neuromyelitis optica spectrum disorders; MS, multiple sclerosis.

References

1. Kampylafka EI, Routsias JG, Alexopoulos H, Dalakas MC, Moutsopoulos HM, Tzioufas AG. Fine specificity of antibodies against AQP4: epitope mapping reveals intracellular epitopes. *J. Autoimmun.* 36(3–4), 221–227 (2011).

2. Gu X, Zhou J, Zhou L, *et al.* Specific binding of antigen-antibody in physiological environments: Measurement, force characteristics and analysis. *Optics and Lasers in Engineering*. 104, 252–258 (2018).

3. Singer SJ, Eggman L, Campbell DH. Physical Chemical Studies of Soluble Antigen-Antibody Complexes. VI. The Effect of pH on the Reaction between Ovalbumin and its Rabbit Antibodies1. *J. Am. Chem. Soc.* 77(18), 4855–4857 (1955).

4. Camilloni C, Bonetti D, Morrone A, *et al.* Towards a structural biology of the hydrophobic effect in protein folding. *Scientific Reports*. 6, 28285 (2016).

5. Lear S, Cobb SL. Pep-Calc.com: a set of web utilities for the calculation of peptide and peptoid properties and automatic mass spectral peak assignment. *Journal of Computer-Aided Molecular Design*. 30(3), 271–277 (2016).

6. Hoh JH, Cleveland JP, Prater CB, Revel JP, Hansma PK. Quantized adhesion detected with the atomic force microscope. *J. Am. Chem. Soc.* 114(12), 4917–4918 (1992).

7. Hoeben FJM, Jonkheijm P, Meijer EW, Schenning APHJ. About Supramolecular Assemblies of π-Conjugated Systems. *Chem. Rev.* 105(4), 1491–1546 (2005).

8. Braden BC, Poljak RJ. Structural features of the reactions between antibodies and protein antigens. *The FASEB Journal*. 9(1), 9–16 (1995).

9. Guo S, Zhu X, Jańczewski D, *et al.* Measuring protein isoelectric points by AFM-based force spectroscopy using trace amounts of sample. *Nature Nanotechnology*. 11(9), 817–823 (2016).

10. Neurath Hans, Greenstein JP, Putnam FW, Erickson JA. The Chemistry of Protein Denaturation. *Chem. Rev.* 34(2), 157–265 (1944).
